# Group mentorship for undergraduate medical students—a systematic review

**DOI:** 10.1007/s40037-020-00610-3

**Published:** 2020-08-20

**Authors:** Elise Pauline Skjevik, J. Donald Boudreau, Unni Ringberg, Edvin Schei, Terese Stenfors, Monika Kvernenes, Eirik H. Ofstad

**Affiliations:** 1 UiT The Arctic University of Norway, Tromsø, Norway; 2 Institute of Health Sciences Education, Faculty of Medicine, McGill University, Montreal, Canada; 3 Center for Medical Education, Faculty of Medicine, University of Bergen, Bergen, Norway; 4 Department of Learning, Informatics, Management and Ethics, Karolinska Institutet, Stockholm, Sweden; 5 Institute of Social Medicine, UiT The Arctic University of Norway, Tromsø, Norway

**Keywords:** Mentor, Undergraduate medical students, Professional development, Mentorship program, Systematic review

## Abstract

**Introduction:**

Mentoring has become a prevalent educational strategy in medical education, with various aims. Published reviews of mentoring report very little on group-based mentorship programs. The aim of this systematic review was to identify group-based mentorship programs for undergraduate medical students and describe their aims, structures, contents and program evaluations. Based on the findings of this review, the authors provide recommendations for the organization and assessment of such programs.

**Methods:**

A systematic review was conducted, according to PRISMA guidelines, and using the databases Ovid MEDLINE, EMBASE, PsycINFO and ERIC up to July 2019. Eight hundred abstracts were retrieved and 20 studies included. Quality assessment of the quantitative studies was done using the Medical Education Research Study Quality Instrument (MERSQI).

**Results:**

The 20 included studies describe 17 different group mentorship programs for undergraduate medical students in seven countries. The programs were differently structured and used a variety of methods to achieve aims related to professional development and evaluation approaches. Most of the studies used a single-group cross-sectional design conducted at a single institution. Despite the modest quality, the evaluation data are remarkably supportive of mentoring medical students in groups.

**Discussion:**

Group mentoring holds great potential for undergraduate medical education. However, the scientific literature on this genre is sparse. The findings indicate that group mentorship programs benefit from being longitudinal and mandatory. Ideally, they should provide opportunities throughout undergraduate medical education for regular meetings where discussions and personal reflection occur in a supportive environment.

**Electronic supplementary material:**

The online version of this article (10.1007/s40037-020-00610-3) contains supplementary material, which is available to authorized users.

## Introduction

Mentoring of medical students has become a prevalent educational strategy, particularly in European and North American medical schools, with the purposes of offering support and guidance, providing a fulfilling student experience and stimulating or sustaining professional development [1, 2]. This method is also utilized to increase students’ understanding of the competencies required of physicians and the professional roles they are to fulfil [3].

While there are multiple definitions of mentoring [4, 5], we recognize that each has its inherent limitations. Thus we have adopted the following—and frequently cited—operative definition: *“A process whereby an experienced, highly regarded, empathetic person (the mentor) guides another (usually younger) individual (the mentee) in the development and re-examination of their own ideas, learning, and personal and professional development” *[6].

The backdrop for establishing mentorship programs in medical education is a number of well-documented stressors that many students face in their learning environments [7–9], influencing professional identity formation, empathy and patient-centered attitudes in a negative way [10–13]. A 2016 study reported that more than a third of medical students have experienced symptoms of burnout [14]. Curriculum overload, high-stake exams, lack of supervision and absence of emotional support characterize many medical students’ daily lives [9, 15]. Measures such as mentorship programs, intended to mitigate these negative influences on students’ formation, are warranted. It has been shown that longitudinal and integrated mentoring can improve psychosocial skills and humanistic attitudes, even when assessed 10 years after graduation from medical school [16].

In 2006, Buddeberg-Fischer et al. identified nine mentorship programs in their review on mentoring medical students and doctors [17]. Most of the programs identified were loosely structured and lacked evaluation strategies. Four group-based mentorship programs were included in the review and the mentees in these programs reported high levels of satisfaction [17]. In 2010, Frei et al. reviewed 14 US mentorship programs; two of the programs provided mentoring in small groups. The authors did not draw any specific conclusions about mentoring in groups [1]. In their 2019 review, Tan et al. suggested smaller groups (of approximately five to eight mentees) when the primary focus is on providing personal support, and larger group sizes when the goal is to discuss professional challenges [18].

Recently published reviews of mentoring in medical education have highlighted key advice for schools considering establishing mentorship programs [19, 20]. However, they do not draw explicit conclusions about mentoring in groups. To the best of our knowledge, no reviews specifically targeting group-based mentorship programs for medical students have been published. Hence, there is a knowledge gap with respect to how group mentorships in medicine are organized and evaluated. Group-based mentorships are resource-heavy and time-consuming; thus, it is essential to explore if they are “worth the hassle” and to identify efficient ways such programs can be structured and evaluated.

Our aim was to identify group-based mentorship programs for undergraduate medical students, and describe their aims, structures, contents and program evaluations. Based on our findings and existing literature, we make recommendations for the organization and assessment of such programs. Quality assessment of the quantitative studies was done using the Medical Education Research Study Quality Instrument (MERSQI).

## Methods

In collaboration with a medical librarian, we conducted systematic searches in the following databases: EMBASE Classic+ (EMBASE 1974 to 2019 July 4), Ovid MEDLINE®, ERIC Database and PsycINFO (to 2019 July 4). The review process was conducted according to the Preferred Reporting Items for Systematic Reviews and Meta-Analyses (PRISMA) guidelines [21]. Tab. 1 presents the search strategy in Medline and Appendix 1 of the Supplementary Online Material summarizes the complete search strategy.

**Table 1:** Search in Medline

Number	
1	Exp Mentors/
2	Exp Mentoring/
3	(mentor^a^ adj3 program^a^).ti
4	(mentor^a^ adj3 group^a^).ti
5	(physician adj3 apprenticeship^a^).ti,ab
6	Education, medical/and education, medical, undergraduate/
7	(Medical adj3 student^a^).ti
8	(Medical adj3 undergraduate^a^).ti,ab
9	1 or 2 or 3 or 4 or 5
10	7 or 8 or 9
11	10 and 11

^a^Indicates truncation

EPS and UR independently conducted the searches between the 1–4 July 2019. Since this study concentrates explicitly on mentoring in groups designed to foster personal and/or professional development, we excluded mentorships with a primary focus on other issues, such as research supervision or career enhancement. We also excluded the grey literature, as one of our inclusion criteria was peer-reviewed papers listed in scientific databases. Tab. 2 presents the PICO analysis describing the selection process in detail.

**Table 2:** Selection criteria

	Inclusion	Exclusion
Population	Undergraduate medical students	Graduate and postgraduate medical students, junior doctors, physicians
Intervention	Description of group-based mentorship programs in undergraduate medical education focusing on professional development Evaluation of the mentorship program, either by mentors or mentees or both	One-on-one mentorship Mentorship programs inadequately described, i.e. lacking details on structure, objectives and/or evaluation Programs aimed at recruiting students to particular specialties or field of interests Programs aimed at medical students who need academic supervision or remediation Programs aimed at under-represented minority medical students
Comparison	Comparison of group-based mentorship programs	
Outcome	Outcomes of mentorship programs on the mentor or mentee Evaluation forms and surveys	
Study design	Peer-reviewed papers	Reviews Conference presentations Commentaries Letters Editorials

**Fig. 1:**
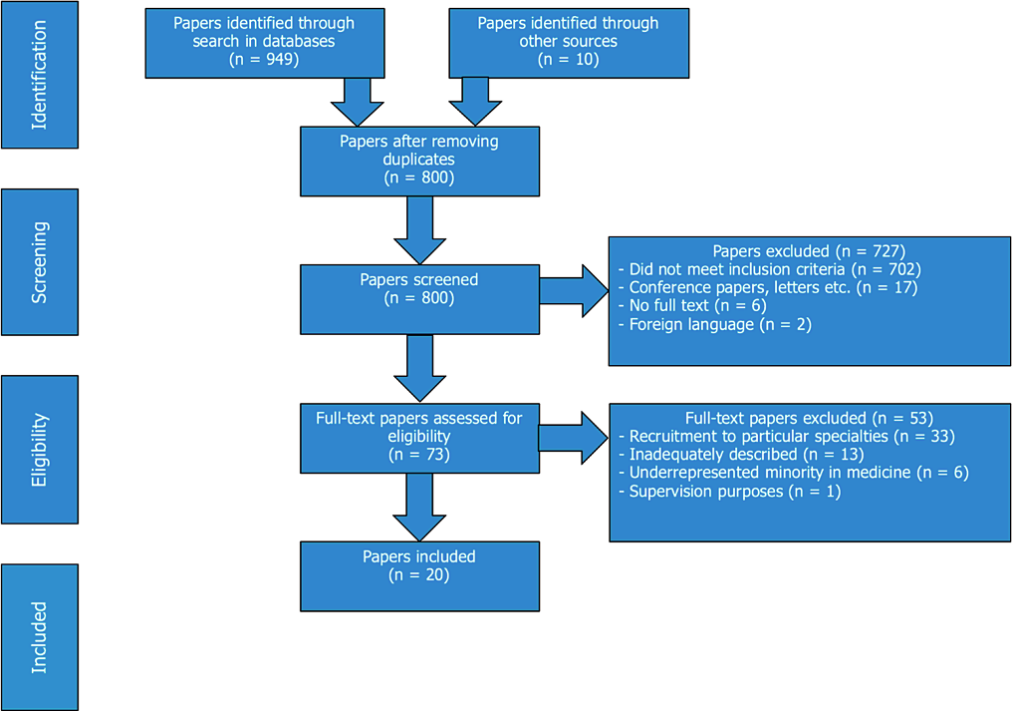
Flow chart

The final search resulted in 949 citations. The authors’ own work and knowledge of the literature resulted in 10 additional records; they were included at this stage for further assessment. After removing duplicates, EPS, UR and EHO screened the titles and abstracts of the remaining 800 records. Fig. 1 provides a flow chart of the review process.

EPS extracted data from each of the 20 included studies using a predesigned system, see Appendix 2 of the Electronic Supplementary Material. The main elements extracted from the studies were the programs’ primary aims, mentorship structure, information on participants and evaluation strategies. Quality assessment of the quantitative studies was performed by EPS and EHO using the Medical Education Research Study Quality Instrument (MERSQI) [22]. The MERSQI items are scored on a scale of 1–3 and summarized to a total score of between 5 and 18 for each study.

We utilized Kirkpatrick’s four-level evaluation model as a framework for categorizing the evaluation approaches used in the studies. Level 1 describes how the participants reacted to the educational program or training (e.g. satisfaction); level 2 assesses the extent to which the participants have learned (e.g. increased knowledge or skills); level 3 examines whether the participants are utilizing their new knowledge (e.g. changed behaviours) and level 4 assesses if the program has a positive impact on the whole organization [23, 24]. This is summarized in Tab. 3.

**Table 3:** Main elements of the programs

Author	Country	Year established	Mentors	Mentees	Mentor-mentee ratio	Longitudinal program	Mandatory	Evaluation	MERSQI (min 5, max 18)	Kirk-patrick levels^c^
Blatt et al. [25]	USA	–	Physicians and psychosocial professionals	MS‑1 to MS‑4	1:4–5 (*2:8–9*)	Y	Y	Y	11	1, 2, 4
Lutz et al. [37]	Germany	2013	Faculty members and near-peers	MS‑1	1:4–5 (*2:8–9*)	N	N	Y	N/A	1, 2
Andre et al. [26]	USA	2006	Faculty members and MS‑4	MS‑1 to MS‑4	1:11 (*4:44*)	Y	Y	Y	6.5	1, 2
Varma et al. [39]	India	2009	Faculty members	MS‑1	1:8–16	–	–	N	N/A	N/A
Kalen et al. [42]	Sweden	2007	Physicians	MS‑1 to MS‑5	1:4	Y	Y	Y	N/A	1, 2, 3
Duke et al. [27]	USA	2015	Faculty members	MS‑3	1:9	N	Y	Y	8	1, 2, 3
Singh et al.^a^ [41]	India	2010	Faculty mentors and near-peers	MS‑1	1:2 (*2:3*)	N	N	Y	7.5	1, 2
Boudreau et al.^b^ [35]	Canada	2005	Physicians and near-peers	MS‑1 to MS‑4	1:3 (*2:6*)	Y	–	Y	N/A	1, 2, 3
Taylor et al. [28]	USA	2006	MS‑2	MS‑1	1:4 (*2:8*)	N	Y	Y	8	1, 2
Fleming et al. [29]	USA	2011	Faculty members	MS‑1 to MS‑4	1:25–28	Y	Y	Y	6.5	1
Bhatia et al. [40]	India	2009	Faculty members	MS‑1	1:2–3	N	N	Y	8	1, 2
Goncalves et al. [43]	Brazil	2001	Physicians	MS‑1 to MS‑6	1:12–14	Y	N	Y	N/A	1
Usmani et al. [44]	Pakistan	2008	Faculty members	MS‑1 and MS‑2	1:10	N	Y	Y	8	1
Elliott et al. [30]	USA	2001	Faculty members	MS‑1 and MS‑2	1:12 (*2:24*)	N	Y	Y	6	1, 2
Macaulay et al. [31]	USA	2003	Faculty members	MS‑1 to MS‑4	1:30	Y	–	Y	6.5	1
Goldstein et al. [32]	USA	2004	Faculty members	MS‑1 to MS‑4	1:6	Y	Y	Y	N/A	1, 2, 3
Scheckler et al. [33]	USA	1985	Faculty members	MS‑1 to MS‑4	One mentor per class	Y	–	N	N/A	N/A
Woessner et al. [38]	Germany	1995	Faculty members	MS from different years	1:12	N	N	Y	6	1
					Median 1:9	Yes: 9 No: 9	Yes: 9 No: 5	Yes: 16 No: 2	Mean 7.4 SD 1.44	

*MS* Medical student; The number following MS denotes the year of the program (e.g. MS‑1 refers to a 1st-year medical student)

*Y* yes, *N* no, – no information

^a^Singh et al. 2010 is the revised version of the mentoring program at the University of Delhi, India, described by Bhatia et al. 2009

^b^Boudreau et al. 2005 is one of three studies identified in the literature search all describing the physician apprenticeship (PA) program at McGill University, Montreal, Canada. Boudreau et al. 2005 describes the program and the assessment of it to such an extent that the two other studies need not be included in the table.

^c^Kirkpatrick levels; Level 1 refers to the level of reaction or feelings by the learners to all factors in an educational program. Level 2 refers to the changes in the learners caused by participation in the program. Level 3 reveals whether or not the program has created a change in the learners’ behavior. Level 4 indicates if the program is effective in meeting the organizational goals

## Results

The 20 studies included describe 17 different group mentorship programs. Three of the studies describe the “Physician Apprenticeship” program at McGill University in Montreal, while two studies describe the mentoring program at the University of Delhi, which was revised in 2010 and is therefore described in two separate papers.

The studies provided, to a various extent, information about the programs’ aims and structure, participants, evaluation and outcomes. MERSQI scores ranged from 6 to 11 (mean 7.4, SD 1.44, [*n* = 11]). Tab. 3 summarizes the main elements of the different mentorship programs, including MERSQI and Kirkpatrick assessments. Greater details regarding aims, structure, content and program evaluation are presented in Appendix 3 of the Supplementary Online Material.

In the following section, we present the findings concerning organization and aims of group-based mentorship programs and identified challenges, mentor characteristics, and evaluation strategies and results.

### Organization and aims of group mentorship programs

The group mentorship programs identified originate from the USA [25–33], Canada [34–36], Germany [37, 38], India [39–41], Sweden [42], Brazil [43] and Pakistan [44]. All programs were initiated after the year 2000, with the exception of the program at the University of Saarland, Germany, established in 1985 [38]. One study did not provide information about the year of establishment [25].

The majority of the programs (*n* = 9) were longitudinal throughout the medical curriculum [25, 26, 29, 31–36, 42, 43] whereas four programs were aimed at first year students [28, 37, 40, 41] and one program at third year students [27]. Two programs ran through both the first and second year of medical school [30, 44]. There was a large variation in meeting frequency, ranging from twice a year [42] to 24 times a year [30]; more frequent meetings appeared to correlate with the use of predetermined topics [30] and specified skills training [28, 32]. Participation was compulsory in nine of the programs [25–30, 32, 42, 44]. The mentor-mentee ratio ranged from 1:2 to 1:30, with a median group size of 9 mentees.

Programs aimed at first-year students focused mainly on providing an immediate support network and early introduction to professionalism [28, 40]. Some studies reported addressing specific themes related to professionalism, such as empathy [27, 30], patient-centeredness [34–36], cultural competence, collaboration, ethical decision-making [30], altruism, honor and integrity, communication, respect and accountability [32]. A key feature in several programs was reflective discussions on professional challenges. Topics ranged from discussing positive role models and unprofessional conduct observed in clinical settings [31], ethical dilemmas, conflicts and dealing with stress [37] to career choice, study strategies and how to plan for life as a medical student [26].

The structures established to achieve aims in professional development differed greatly. The two following examples illustrate the variation: the medical students at the Karolinska Institute [42] discussed their own development with their physician mentor, using a self-assessment form based on the CanMEDS framework for the physician’s professional roles and competences [45]. Furthermore, each group watched videos focusing on psychological and ethical aspects of physician-patient interactions. In contrast, first-year students at the Alpert Medical School were offered mentoring by second-year students, to foster the students’ professional development and skills in medical interviewing and physical examination [28].

### Who are the mentors?

Faculty members or experienced physicians acted as mentors in almost all programs [25, 27, 29–33, 38, 39, 42–44]. Some programs provided dual mentoring; frequently, the mentor pair consisted of a faculty member and a senior medical student [26, 35, 37, 41]. One program was based solely on peer-mentoring, with mentors being second-year and mentees first-year students [28].

In some programs [28, 37], the mentors were volunteers. Only four studies [29, 31–33] reported on financial compensation, which ranged from 12,000 USD [33] to 30,000 USD per year [31]. Furthermore, four studies reported on the amount and quality of faculty development for the mentor role, describing that the mentors were invited to workshops [25, 28, 37], seminars [42] and supervisory meetings [43] in order to prepare for group sessions and share experiences with colleagues.

### Evaluation strategies and results

All except three programs conducted some form of evaluation. The majority of programs (*n* = 8) were evaluated by questionnaires [26, 28–31, 38, 40, 44]: four invited both mentors and mentees to participate [26, 28, 38, 40], one was answered by mentors only [44]. The response rates among mentees varied from 28% [26] to 68% [31]. Three studies conducted interviews to collect data for an evaluation, either individual or in focus groups [37, 42, 43]. Two programs were evaluated using a mixed-method design [25, 35]. Finally, three programs were evaluated using other methods such as qualitative statements from mentors [33], the Groningen Reflection Ability Scale (GRAS) and Jefferson Scale of Empathy (JSE) [27], results from Mini-Clinical Evaluation Exercise (Mini-CEX) and Objective Structured Clinical Examination (OSCE) [32].

Using Kirkpatrick’s model of evaluation, most evaluations report findings consistent with level 1 (reaction/satisfaction) and 2 (learning, based on self-reports). Five studies provide information about how the group-based mentorship program induced changes in student behaviour or practices (level 3) or organizational benefits (level 4). The program at the University of Texas San Antonio [26] was the only one to use annual questionnaires for evaluation. The students reported significant year-to-year improvements, and post-hoc analysis showed that the program had increased students’ undergraduate medical school satisfaction.

The program at Witten/Herdecke University was evaluated using semi-structured focus group interviews with students and semi-structured individual interviews with mentors and co-mentors. Some students did not seem to perceive any positive outcomes on their professional development or understand why improving their performance as physicians was connected to their abilities to reflect on and discuss personal and professional challenges. Other students mentioned improved abilities to partake in discussions of a reflective nature, thus enhancing the comprehension of themselves and others [37].

The Physician Apprenticeship at McGill University was evaluated by conducting a longitudinal, mixed-methods study. The design was a case study, consisting of three physician apprenticeship groups (a total of 24 medical students and three mentors) followed over four years. The authors concluded that a long-term mentoring program can contribute to building and maintaining a professional identity among medical students and to reaffirming the professional identity of mentors [34–36].

The program established at Drexel University is one of the few that evaluated its effects on students’ competence. Students were assessed before and after the program by mapping their abilities to engage in self-reflection and perceived empathy using the Groningen Reflection Ability Scale (GRAS) and Jefferson Scale of Empathy (JSE). The program increased students’ reflection abilities and may have contributed to the preservation of empathy. GRAS scores increased significantly (*p* < 0.001) in both genders, while JSE scores were unchanged [27].

Overall, most of the studies reported positive effects of group mentoring. Students highlighted increased personal and social support [30, 31, 33, 35], improved student satisfaction and professional growth [26, 29, 30]. Mentors reported personal and professional gain [35, 37, 44], increased skills in communication and feedback [40] and felt gratified to see the students develop professionally [35, 37, 38, 44].

### Challenges for group-based mentorship programs

Some of the studies described barriers to well-functioning mentoring. In evaluating the mentorship program at Sao Paolo University in Brazil, many mentors expressed frustration because of the students’ low attendance or absence. Furthermore, they experienced doubt in dealing with the initial expectations about the mentoring role [43]. Both at Bahria University in Pakistan [44] and Sao Paulo University [43], some of the mentors felt burdened at times as mentoring was an additional and time-consuming assignment. The students identified various impediments to positive interpersonal communication, including lack of reliability, breaking confidentiality rules and disrespect in the groups.

At the University of Delhi, about one third of the mentorship groups never met during the academic year, mentees were often reluctant to contact the mentors, and finding the appropriate time for all parties was described as a common challenge [40]. Various other barriers were reported, including: technology issues, logistics, a lack of ‘personal chemistry’ in the group and time constraints [27].

## Discussion and recommendations

Our systematic review reports on the nature of group-based mentorships in medical schools located in seven different countries. The programs included in this review had similar overall aims (personal and professional development and student support). However, we found large variations in the way they were organized. This may reflect differing interpretations of professionalism among universities and suggests that there are several ways to foster professional development.

A key element of transformative learning in professional development is partaking in reflective discussions with others [46]. Medicine is teamwork, hence communication skills and reflective discourses in group settings are essential parts of being a physician. Whilst the intimacy of one-on-one mentoring may facilitate coaching on the personal aspects and unique vulnerabilities of an individual student’s educational experience, a group setting can provide a framework that offers rich possibilities for relationship building. This format provides an avenue for peers of varied backgrounds and resources to share experiences and to reflect on social interactions and relational skills [47].

In the following discussion, we draw upon the institutions’ experiences with group-based mentoring, as presented in the 20 studies, and explore the essential factors for well-functioning group mentorship programs. The majority of the studies provided sufficient information on mentorship structures and evaluation strategies and have permitted us to propose a set of recommendations for group-mentorship programs. These are presented in Tab. 4.

**Table 4:** Recommended features for mentorship programs

The mentorship program should be longitudinal throughout the medical education
Mentorship activities should be designed to align with the overall curriculum
The program should be mandatory
Mentors should be (experienced) physicians, either alone or in pairs, and may be accompanied by a student mentor
A small financial reward or promotion for mentors may reduce “wear and tear”
Mentors should be empowered by introductory courses, frequent mentor gatherings or workshops and faculty support

### Optimal organizational features

Most of the identified programs were longitudinal. Assessment of one of the shorter programs reported that both mentees and mentors wished their program were longer in duration [38]. The students at the Witten/Herdecke University stated that integrating the group mentoring into the entire curriculum (i.e. longitudinal program) was seen as “essential in experiencing the relevance of reflection” [37]. In a longitudinal program, the mentoring relationship can evolve over several years, hence it can facilitate openness and reflective discourses. Moreover, group dynamics may take time to establish and require investment in a trustworthy learning environment. We therefore suggest that longitudinal group mentorship programs focusing on professional development are preferable to shorter programs limited to a single or a few years.

We found large variation in meeting frequency, and more frequent meetings appeared to be correlated with groups having predetermined topics [30] and skills training [28, 32]. We propose a minimum of two meetings per semester, with higher meeting frequencies both in the beginning of medical school and during clinical rotations. This has been shown to be important in providing an immediate network of safety and support and to debrief students’ clinical and emotional experiences [15].

Recent studies propose that mentorship activities should be designed to fit the overall curriculum [18, 20]. If a mentorship program is loosely attached to other teaching and learning activities, it may become a competing activity that can be easily ignored. Mandatory attendance might be one mechanism to meet this challenge. A frequent complaint from mentors was that mentees did not attend the groups consistently in voluntary programs [40, 43].

Mandatory group meetings not only ensure mentee participation, it also signals the importance of group mentoring as a meaningful part of the curriculum. In fact, none of the programs in this review reported that a mandatory approach was considered negative. Based on our findings, a mandatory approach to group mentorship seems preferable. It is important, however, that compulsory teaching activities are adequately resourced and continuously evaluated to ensure a high standard [20].

### Who should mentor medical students in groups?

The majority of the studies reported that either physicians or faculty members fill the roles of mentors. If the mentorship aim is to foster professionalism, it may be reasonable to recommend experienced physicians over near-peer mentoring by medical students. However, our findings indicate that a combination of a physician mentor assisted by a senior student can work really well [26, 35, 37]. In evaluating the revised program at the University of Delhi [40], nearly all faculty mentors and mentees appreciated the contributions of the co-mentors [41]. The involvement of experienced student mentors can be preferable as it will maintain desirable mentor-mentee ratios, especially in medical schools with large classes where it may be difficult to recruit enough physician mentors.

With regards to incentives for mentors, our findings do not indicate that they are essential to motivate mentors. For instance, the group mentorship program at the University of Saarland is described as well-functioning and popular with both mentees and mentors, even without faculty support, incentives and mandatory participation [38]. However, for the recruitment and sustainability of a motivated mentor force, a small financial reward or promotion may reduce “wear and tear”.

Nimmons et al. recommended that mentors should receive guidance in the requirements of the role and in delivering effective feedback to mentees [20]. Faculty development and administrative support to mentors in one of the identified programs was described as a key element [26]. Many mentors at Sao Paolo University experienced doubt concerning the expectations of the mentor role and its tasks [43]. We suggest an approach to empower group mentors: firstly, every mentor should participate in an introductory workshop where the program aims and methods to achieve these aims are emphasized [28, 37, 42, 43]. Secondly, mentors should have the possibility to attend frequent mentor gatherings to facilitate debriefing and reflective discussions [42, 43].

### Program evaluation

In evaluations using a quantitative design, the response rates varied considerably. Low response rates (<50%) increase the risk of selection bias and hamper external validity, which was the case in some studies [26, 27], while response rates were not reported in others [29, 30, 38]. One of the programs used the four-level Kirkpatrick model for evaluation [25]. Only a few studies reported on barriers to well-functioning mentoring; there is a need to address such challenges in future studies.

The two most informative evaluations were both conducted using mixed methods [25, 35]. Mixed-methods design may be advisable for researchers who want to describe and assess group mentorship programs in the future, in order to collect comprehensive data. Additionally, case-studies as described in some of the included studies [27, 37] can be recommended as an approach to provide more in-depth knowledge concerning educational strategies [48].

### Limitations

A significant limitation of this study is the variety of approaches used to evaluate the mentorship programs. Lack of uniform terminology and diverse evaluation strategies, especially non-validated methods of assessment, makes it challenging to compare outcomes of mentorship programs [49]. There is a need for more research-based evaluation designs of group mentorship programs, particularly to learn more about the effects of programs at Kirkpatrick’s level 3 and 4.

The studies assessed with MERSQI in this review ranged from 6 to 11 (mean 7.4, SD 1.44, *n* = 11). Most of the studies used a single-group cross-sectional design conducted at a single institution, hence yielding a low score. Furthermore, none of the studies reported validity of evaluation instruments. This, combined with low or non-reported response rates, resulted in mostly low MERSQI scores for studies using quantitative assessments. This makes it difficult to draw robust conclusions from most of the identified studies.

Given our decision not to include the grey literature, we may not have benefited from the experience of group mentorships that have been implemented but not reported on in the peer-reviewed literature. Future studies should consider performing an adjuvant search in the grey literature.

Our findings indicate that the establishment of mentorship programs for medical students, including group-based programs, is a trend worldwide. However, when considering the absolute number of medical schools, particularly in continental countries, there is reason to believe that the 17 group-based programs identified in this review represent a small percentage of existing programs.

## Conclusion

Group mentoring as an educational strategy for medical students holds great potential. We identified 17 different mentorship programs in seven countries, and the evaluation data are remarkably supportive of mentoring medical students in groups. However, the scientific literature on this emergent genre is sparse and the quality of publications is modest. Our findings indicate that group mentorship programs benefit from being longitudinal and mandatory throughout undergraduate medical school, and that mentorship organizers must pay close attention to ensuring the quality of the program through curriculum alignment, the support of mentors and continuous evaluation to keep the program on track.

## Caption Electronic Supplementary Material

Appendix 1: Search terms

Appendix 2: Data extraction

Appendix 3: Detailed characteristics of group-based mentorship programs (listed by year of publication)
