# Supplementary figures and images for: Group mentorship for undergraduate medical students—a systematic review
Source: Perspect Med Educ. 2020 Aug 20;9(5):272–80. doi: 10.1007/s40037-020-00610-3 (PMC7550430; doi:10.1007/s40037-020-00610-3)

**Appendix 1 Search terms**


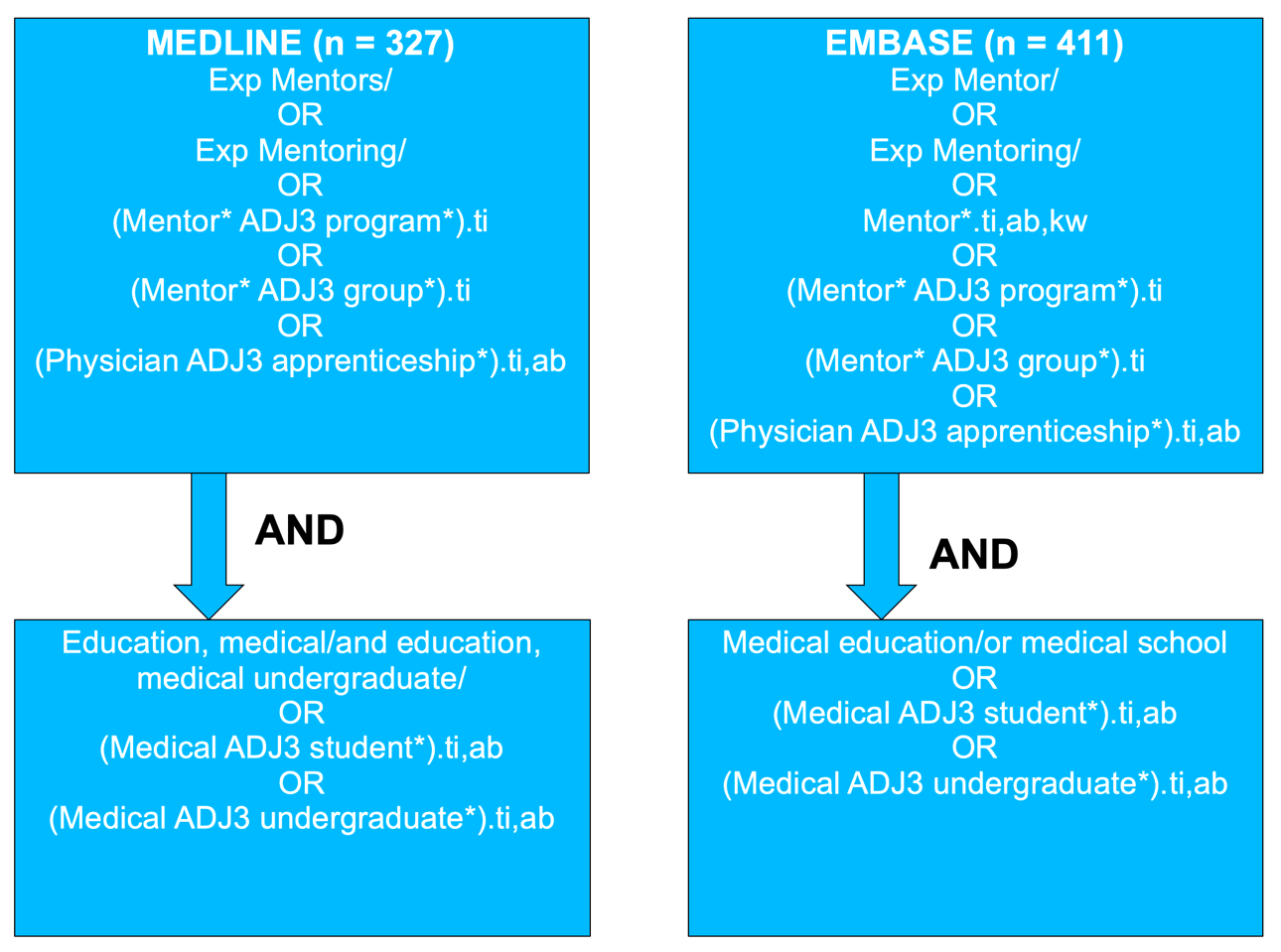

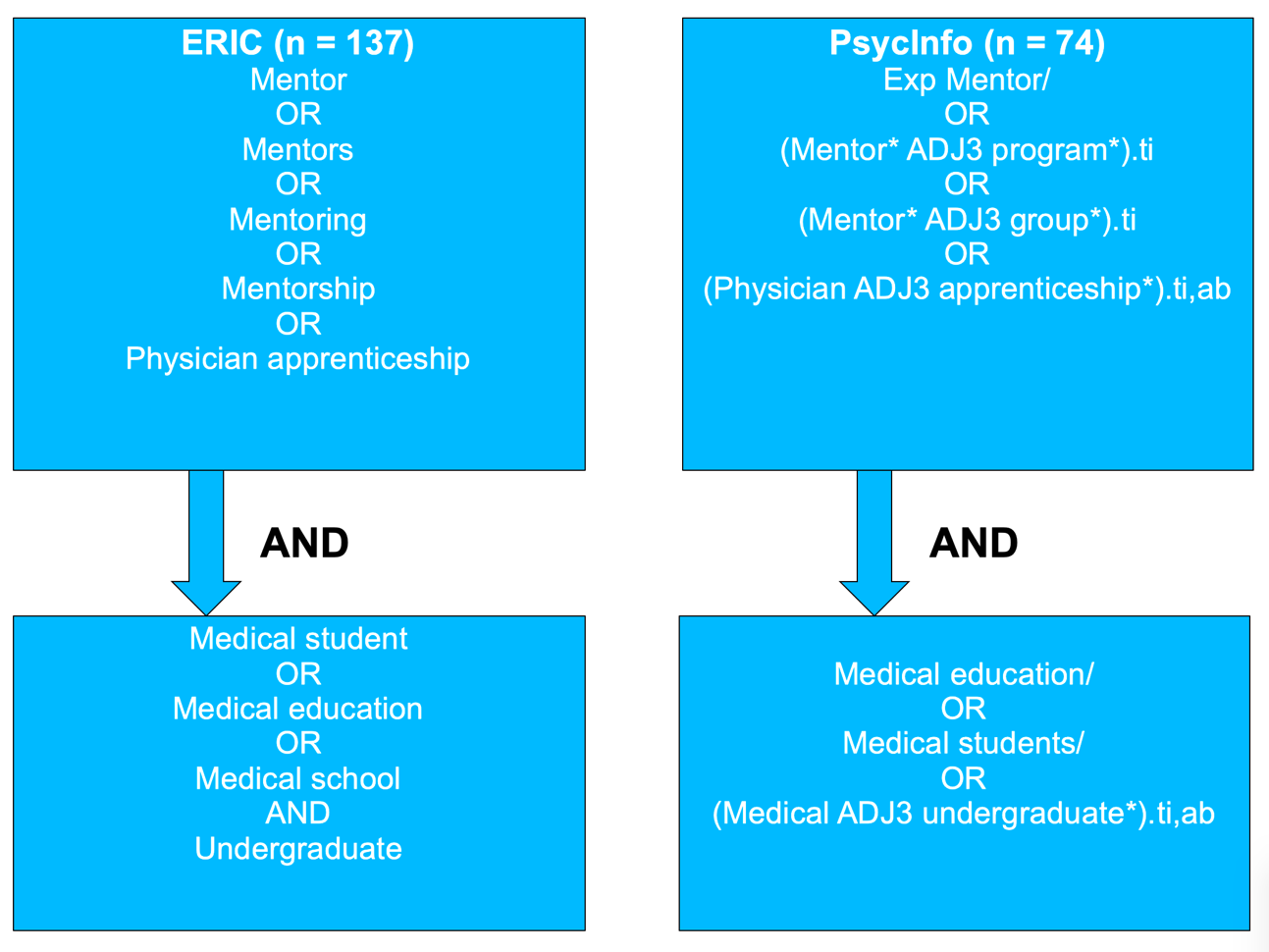

Supplement: Supplementary file 1 — Appendix 1: Search terms [file 40037_2020_610_MOESM1_ESM.docx]
