# Supplementary material for: Group mentorship for undergraduate medical students—a systematic review
Source: Perspect Med Educ. 2020 Aug 20;9(5):272–80. doi: 10.1007/s40037-020-00610-3 (PMC7550430; doi:10.1007/s40037-020-00610-3)
Supplement: Supplementary file 2 — Appendix 2: Data extraction [file 40037_2020_610_MOESM2_ESM.docx]

**Appendix 2 Data extraction**

1. Year published
2. University, city, country
3. Year established
4. The programs’ main objective
5. Mentorship structure
   1. Part of a course
   2. How many mentees per mentor
   3. Number of meetings, topics discussed
   4. Compulsory or non-compulsory
   5. Incentives or compensation for mentors
6. Participants
   1. Mentees
   2. Mentors
7. Evaluation strategy
   1. Qualitative, quantitative data or both
   2. Mentees, mentors or both
   3. Response rates
   4. Results
