# Supplementary material for: Group mentorship for undergraduate medical students—a systematic review
Source: Perspect Med Educ. 2020 Aug 20;9(5):272–80. doi: 10.1007/s40037-020-00610-3 (PMC7550430; doi:10.1007/s40037-020-00610-3)
Supplement: Supplementary file 3 — Appendix 3: Detailed characteristics of group-based mentorship programs (listed by year of publication) [file 40037_2020_610_MOESM3_ESM.docx]

**Appendix 3 Detailed characteristics of group-based mentorship programs (listed by year of publication)**

Legend:

MS = Medical student

The number following MS denotes the year of the program (e.g. MS-1 refers to a 1^st^-year medical student).

| **Blatt et al., 2018. The George Washington University School of Medicine and Health Sciences, USA.** | | | |
| --- | --- | --- | --- |
| **Aims**   - To encourage student professional identity formation in the terms of the biopsychosocial model - To support student development into physicians | **Structure and content**  Mentoring as part of the Professional  Development (PD) Course.  Each medical student class were divided into six learning communities (LCs). In each LC, eight faculties served in pairs as mentors for four small groups of students, thus two mentors per group of eight to nine medical students.  In total, 22 medical doctors and 22 psychosocial professionals mentored 180 medical students per class through the four years of medical school.  Mentors monitor students’ development through four sorts of activities that comprised the content of the PD Course:   - Small group PD sessions, monthly - Portfolio reflection session, monthly - Professional Progress Assessments, end of semester; mentors meet with students one-on-one - Reflection Rounds - end of semester | **Participants**  Mentees: MS-1 to MS-4  Mentors:   1. Medical doctors 2. Psychosocial physicians (volunteers from the community and psychiatrists) | **Evaluation strategy and results**  Evaluation of the faculty program for mentors by using a mixed-method design (quantitative; mentor and student surveys and qualitative; open-ended survey questions and focus group) data from mentors. The quantitative results were analyzed using a modified four-step Kirkpatrick model.  Results:  High response rates;  Mentors: Ranging from 84% (37/44) to 66% (29/44)  Mentees: 68% (122/180)   - Mentors rated the program highly (mean scores ranged from 3.27 to 4.16) and found the workshops useful (3.81) - Mean student rating of the mentors was 4.34 (max 5.0) - Three main themes were identified from analyzing the qualitate data; 1) Interprofessional faculty communities developed through observation, collaboration, reflection and dialogue; 2) Humanistic mentors created safe environments for student engagement and 3) Engaging in interprofessional humanistic communities of practice expanded mentors’ personal and professional identities |
| **Lutz et al., 2017. Witten/Herdecke University (WHU), Germany. Mentorship program since 2013.** | | | |
| **Aims**   - To support personal and professional development through reflection and discussion | **Structure and content**  Small groups of eight to nine students met with mentors and co-mentors to reflect on and discuss personal professional topics they encountered in medical school. Topics included what it means to be a physician, how to encounter ethical dilemmas, conflicts and stress in medical education.  Both mentors and co-mentors were volunteers.  The selection of mentors was done according to their teaching reputation. Both mentors and co-mentors were trained in a 90-minute workshop regarding their roles in the program. | **Participants**  Mentees: MS-1  Mentors: Faculty teachers | **Evaluation strategy and results**  Individual, semi-structured interviews with eight mentors and one co-mentor and semi-structured focus group interviews with 14 students.  The aim of the study was to advance the understanding of whether reflective discourse on professional challenges in groups is considered as useful for professional development; and which factors influenced how students engaged in the groups.  Results:   - The students’ attitudes towards reflective discourse professional challenges were diverse - Some highly valued the program and some expressed aversive attitudes – the reasons were unclear goals and benefits, interpersonal problems within the groups hindering development and intrapersonal issues - Several suggestions on program setup were made; explaining the program thoroughly, setting expectations and integrating the reflective discourse in a meaningful way into the curriculum, obliging participation without coercion, developing a sense of security, trust and interest in each other within the groups, randomizing group composition and facilitating group moderators as positive peer and faculty role models and as learning group members. - Both mentors and co-mentors reported personal gain |
| **Andre et al., 2017. The University of Texas San Antonio School, United States. Mentorship program since 2009.** | | | |
| **Aims**  To enhance the professional development of medical students by   - fostering relationships - assisting students to make informed decisions about their careers | **Structure and content** “Mentors in Medicine” (MiM) was established in 2009 as a supplement to the existing student-advising program (“Veritas”) with clinical faculty.   Students were admitted randomly to one of 20 Veritas groups. Each group had one faculty mentor, three MiM and 44 medical students. The groups were further divided into subgroups, meeting at least once a month.  Participation was mandatory.   3^rd^ year students were eligible to apply for the role as MiM and were chosen by a committee of Veritas faculty and students.  MiMs orchestrated group meetings, including assessments after each meeting. A longitudinal MiM elective program aimed to develop students’ mentorship, leadership and communication skills. | **Participants**  Mentees: MS-1 to MS-4  Mentors:   1. Clinical faculty   and   1. MS-4 | **Evaluation strategy and results**  Annual survey data of all students as well as student mentors to evaluate program effectiveness to assess the impact of the MiM initiative as an adjunct to the Veritas program.  Results:  Students as mentees   - All-student survey:   Low response rates; 28.9% (127/439) responded in 2011, 39.5% (183/463) in 2012, 45.0% (198/440) in 2013, and 36.8% (162/440) in 2014.  Overall, students reported significant year-to-year improvements in their Veritas experiences from 2011 to 2014.  Students as mentors   - MiM survey:   High response rates; 70.5% (43/61) in 2012, 70.7% (41/58) in 2013, and 77.4% (48/62) in 2014.  One hundred percent of MiMs in 2012 (43/43) and 2013 (41/41) and 91.7% (44/48) in 2014 said they would still volunteer to be a MiM after having served as one.  Most frequent responses regarding successful Veritas group needs included; participation among all members; communication, and faculty member support. |
| **Varma et al., 2016. Pramukhswami Medical College, India. Mentorship program since 2009.** | | | |
| **Aims**   - To encourage students professional and personal development | **Structure and content**  Each group consisted of 8-16 mentees and one mentor. The groups met face-to-face two to three times per year. These meetings were prearranged.  Some mentors also met their mentees to discuss personal challenges.   Participation was mandatory.   The major focus in the meetings was on issues other than personal and professional growth. Mentors recorded problems reported by the students and forwarded to the office of the Dean for perusal.   The faculty mentors were volunteers. | **Participants**  Mentees: Entry-level students. As the students progressed to the next phase of the undergraduate program, different mentors were assigned to them Mentors: Faculty members | **Evaluation strategy and results**  No systematic evaluation |
| **Kalen et al., 2015. Karolinska Institutet, Sweden. Mentorship program since 2007.** | | | |
| **Aims**   - To facilitate professional and personal development; focusing on non-medical skills | **Structure and content**  The groups consisted of four medical students and one mentor. Groups of four students met their mentor on a ‘workshop day’ once a term. The mentoring program was longitudinal through all 5.5 years.  Participation was mandatory.   - One-on-one   The students’ own development was discussed individually with the mentor, using a self-assessment form based on the CanMEDS framework. Students set up goals and developed an action plan for improvement in these roles.   - The group   watched videos focusing on psychological and ethical aspects of the interaction between the physician and patient, with increasing complexity across terms. This was followed by a discussion with peers and the mentor about how to handle such situations professionally.   The mentoring program also offered opportunities for the students to visit the mentor in connection with their clinical work. | **Participants**  Mentees: MS-1 to MS-5  Mentors: Physicians working in the health care system, some being clinical teachers or supervisors | **Evaluation strategy and results**  Individual, semistructured interviews of 16 mentees from each of the term 2, 4, 6 and 8. The aim of the study was to explore how formal longitudinal mentoring can contribute to medical students’ professional development.  Results:  The analysis resulted in three main themes;   - Integration with one´s future role as a physician - Experiencing clinical reality with the mentor creates incentives to learn - Towards understanding the professional competence of a physician |
| **Duke et al., 2015. Drexel University College of Medicine, United States. Mentorship program since 2012.** | | | |
| **Aims**   - Professional development; self-reflection and empathy | **Structure and content**  Group mentoring in “Virtual Reality”  The course “Professional Formation Curriculum” was revised and expanded in 2012. In concurrence with the revision, virtual classrooms were developed using Google+ Hangout social networking technology.   Students remained in the same group throughout the course, and faculty facilitators changed each year.  The groups met virtually every 8-12 weeks in a 75-min group session. Additionally, the students wrote blogs or short narratives for each session.  Participation was mandatory.   Faculty facilitators were given separate training and facilitator guides for each session; these defined goals, format and proposals for improving virtual facilitation. | **Participants**  Mentees: MS-3  Mentors: Faculty members | **Evaluation strategy and results** Groningen Reflection Ability Scale (GRAS) and Jefferson Scale of Empathy (JSE), and a required anonymous end-of-course online feedback from mentees. The purpose of the study was to assess empathy and self-reflection among the students before (Time 1) and after (Time 2) the course.  Results:  High response rate Time 1; 96% both GRAS and JSE (249/259).  Low response rate Time 2; 49% GRAS (129/259) and 48% JSE (125/259).  The GRAS scores among medical students of both genders significantly increased (p < 0.001), suggesting that the mentoring program promoted the students’ abilities to reflect. The Jefferson Scale of Empathy (JSE) scores were unchanged.  70% of the respondents (n = 168) agreed or strongly agreed that the mentoring program enhanced their understanding of the obstacles they and their peers face during medical school.  Most of the negative feedback focused on technology challenges of the program. |
| **Boudreau et al., 2014. McGill University, Canada. Mentorship program since 2005.**  **Shevell et al. (2015) and Steinert et al. (2010) describe the same program.** | | | |
| **Aims**   - To provide a safe and caring environment where students are encouraged to discuss issues arising out of the educational experience; - To guide students in becoming reflective and patient-centred - To assist in the transition from layperson to physician | **Structure and content**  The mentoring program was called the “Physician Apprenticeship” (PA). Its mentors were entitled “Osler Fellows”.   The PA-groups consisted of six medical students and two mentors. Groups remained stable through four years and met approximately five times per year.   Osler Fellows were recruited on the basis of their reputation, recommendations and student nominations. | **Participants**  Mentees: MS-1 to MS-4  Mentors: One physician (“Osler Fellow”), and two 3^rd^- or 4^th^-year medical students in the first two years of the group (“Osler co-leader”) | **Evaluation strategy and results**  Longitudinal, mixed-method case study. The evaluation methods were document analysis, focus group interviews, semistructured individual interviews, observation of group meetings, field notes, written and online questionnaires. Audio recordings of reflections from mentors were solicited during the first year of the apprenticeship.  Results:   - Increased self-reflection - Increased personal support - The mentoring group was considered important for both students and mentors - The Osler Fellows found personal and professional rewards in mentoring   Mentors reported a sense of honor and a feeling of privilege in accompanying students (mentees) on their journey of discovery. They reported a joy of working with students and a desire to make a difference. |
| **Taylor et al., 2013. Alpert Medical School, United States. Mentorship program since 2006.** | | | |
| **Aims**   - To improve medical interviewing and professional skills | **Structure and content**  Mentoring as part of the 2-year required preclinical course (“Doctoring”). The mentoring program was entitled “Teaching Academy” (TA).  Each TA fellow was assigned to work with four 1^st^-year students, thus each small group of eight students had two TA fellows. Each TA-fellow met with two students regularly three to four times per semester and before the practical exam.  Participation was mandatory.  The course combined training and assessment in medical interviewing, physical examination and professional development. The TA Fellows volunteered their time to mentor MS-1 and were not financially compensated. | **Participants**  Mentees: MS-1  Mentors: MS-2 systematically selected by course faculty and trained as “TA Fellows” | **Evaluation strategy and results**  Questionnaire (qualitative and quantitative data from mentors and mentees).  Results:  Mentees:  High response rate; 64 % (60/95)   - A significant increase of confidence in their ability to perform medical interviews and physical examinations (p < .001 for each)   Mentors:  High response rate; 100 % (24/24) and 79% (19/24)   - A significant increase of confidence in their ability to provide feedback (p < .001) - Improvement of clinical skills |
| **Fleming et al., 2013. Vanderbilt University School of Medicine, United States. Mentorship program since 2011.** | | | |
| **Aims**   - To contribute to students’ professional development; focus on medical humanism, leadership, research and society | **Structure and content**  Mentoring as part of the four-year “College Colloquium” course.   The class met for 30 minutes and were then divided into groups of 25-28 students meeting for 1½ hours. In addition, students were required to submit multiple written reflections each semester. Students prepared for sessions via assessment of allotted readings that were intended to challenge their knowledge, assumptions, and beliefs.   Participation was mandatory.  Faculty members were selected as mentors to teach the medical humanities and lead sessions dedicated to student professional development in the areas of leadership, research, and service-learning.  Recruitment criteria were “creativity, enthusiasm, accountability, good judgment, and a positive mindset” in addition to “a background in teaching and advising at the undergraduate medical education level.”  The College Mentors (CMs) received a 0,3 full-time equivalent salary support. The project was built on close-knit cooperation between faculty, student organizations and university leaders. | **Participants**  Mentees: MS-1 to MS-4  Mentors: College Mentors (CMs) | **Evaluation strategy and results**  Questionnaire comparing 2004 (pre-colleges) and 2012 (colleges fully implemented).  Results: Respondents; 245 first- through fourth-year students   - Improved student satisfaction; both faculty mentoring from 75.6% in 2004 to 87.5% in 2012 and career planning from 67.5% in 2004 to 82.9% in 2012 (preliminary data) - > 91% of the responding first- through fourth-year students indicated that the colleges contributed meaningfully or somewhat meaningfully to their Vanderbilt experience - Respondents explained CMs as approachable (92%), accessible (91%), and responsive (93%) |
| **Bhatia et al., 2013. University College of Medical Sciences, University of Delhi, India. Mentoring program since 2009.**  **Singh et al. (2014) describe the same program after a revision.** | | | |
| **Aims**   - To provide 1^st^-year medical students with an immediate support network - To encourage the mentees to reach their full potential by sharing knowledge and experience, and providing emotional support and encouragement | **Structure and content**  Fifty-five volunteer faculty mentors were assigned two-three students each; 150 1^st^-year students in total.  Participation was elective.  The faculty mentors and students were advised to visit the website of the Medical Education Unit to find details of the expected roles of mentors and mentees. | **Participants**  Mentees: MS-1  Mentors: Faculty members | **Evaluation strategy and results**  Questionnaire (qualitative data from mentors and mentees).  Results:  Response rates; mentees 52% (78/150) and mentors 52,7% (29/55)   - Benefits of mentoring:   Improved personal, academic and emotional qualities, both for mentors and mentees. Mentors reported development of communication and affective skills as the prime achievements.   - Barriers to mentoring:   Being unable to find common time to meet; no contact was made in about one-third of instances; mentors assumed that the responsibility was on the mentee. Mentees were often hesitant about talking to faculty members. |
| **Gonçalves et al., 2012. Faculdade de Medicina da Universidade de São Paulo, Brazil. Mentorship program since 2001.** | | | |
| **Aims**   - To offer a mentor who will follow the medical students throughout the course, with regard to their professional and personal development - To stimulate sharing of experiences | **Structure and content**  Mentoring as part of “Programa Tutores (FMUSP)”.  The program was implemented in 2001 for all 1,080 students. Each mentor was responsible for 12-14 mentees.  Participation was elective. | **Participants**  Mentees: MS-1 to MS-6  Mentors: Physicians involved in the educational context | **Evaluation strategy and results**  Semi-structured interviews with 14 mentors; open-ended questions regarding their motivations, difficulties, support resources and changes over time.  Results:   - Many mentors recognized doubts and difficulty in dealing with the initial expectations about the role and the mentoring tasks - Over time, the greatest difficulty perceived by most of the mentors was the students’ low attendance - Some mentors did not identify any difficulty in being a mentor |
| **Usmani et al., 2011. Bahria University Medical and Dental College Karachi, Pakistan. Mentorship program since 2008.** | | | |
| **Aims**   - To encourage students’ development at an academic and personal growth level | **Structure and content**  Each mentor was responsible for approximately 10 mentees. The mentoring method was done in two stages every fortnight. The schedule was a part of the 1^st^ and 2^nd^ -year time table and a slot was dedicated to this process to ensure that the students and teachers were free to participate easily. During stage one the students were spoken to in focus groups which had approximately 10 students. In the second stage mentees were called one by one and this interaction was personal and confidential.  Participation was mandatory.   The mentors were involved in all activities related to their mentees and maintained their records in the form of a portfolio. The portfolio was to make both mentor and mentee aware of the mentees’ development. | **Participants**  Mentees: MS-1 to MS-2  Mentors: Faculty members | **Evaluation strategy and results**  An anonymous data collection tool distributed to 22 mentors constructed with both closed ended and open-ended questions.  Results:  High response rate; 100% (22/22)   - The majority of mentors deemed themselves as "good" (55%) or "satisfactory" (45%) mentors - 73% (yes+sometimes) considered themselves confident about their role and ability to provide full guidance to the students, even to the extent of protecting them - All of the mentors expressed a sense of personal satisfaction and fulfillment in observing their mentees’ success - The mentor-mentee relationship was considered valuable to both parties |
| **Elliott et al., 2009. University of Southern California, USA. Mentorship program since 2001-2002.** | | | |
| **Aims**   - To create a community and a social context in order to support the learning of professionalism - To build professional identity - To improve students’ empathy, cultural competence, collaboration, and ethical decision-making | **Structure and content**  Mentoring as part of the longitudinal course “Professionalism and Practice of Medicine” (PPM).  Each learning community was made up of 24 students. One faculty mentor was assigned per learning community.   Participation was mandatory.  Learning activities proceeded in variable formats. Many of the sessions started with a lecture hall presentation. Student discussion then occurred in four subgroups of six students each. This was followed by a wrap-up discussion where the entire learning community of 24 students were reconvened. There were 24 meetings in Year 1 and 16 meetings in Year 2. Many of the sessions in second year were student led. The faculty mentors served as facilitators and guides to the student leaders. Faculty members were expected to act as role models for medical students and to provide guidance, support and advice. | **Participants**  Mentees: 1^st^- and 2^nd^-year medical students at pre-clerkship level  Mentors: Faculty members who are selected on the basis of their teaching expertise | **Evaluation strategy and results**  Evaluation of course and faculty members using questionnaires with both quantitative and qualitative elements. The former used a 5-point Likert scale for each item. These were administered twice in Year 1 and once in Year 2.  Results:   - Better skills related to the course's objectives; specifically on the following: communication skills; social and community context of health care; ethical judgement; self-awareness, self-care and professional growth; and professionalism - The mentors were considered as role models and resources throughout medical school and in future careers |
| **Macaulay et al., 2007. Columbia University College of Physicians and Surgeons, USA. Mentorship program since 2003-2004.** | | | |
| **Aims**   - To advice, guide and support students in their academic and professional development and extracurricular activities | **Structure and content**  Mentoring as part of the “Avisory Dean (AD) Program”. One mentor, named “AD”, was assigned to 30 medical students.  The mentees were introduced to their mentor during the first week of medical school, following weekly meetings in September and every two weeks thereafter, and second-year student meetings occur  every two weeks. These meetings with the ADs occurred in both structured and informal sessions. Each AD was also available for help or advice via e-mail, phone, and regular office hours.  Each AD committed to spend 20% of his or her  time to mentoring, for which the dean’s  office provided $30,000 per year. | **Participants**  Mentees: MS-1 to MS-41  Mentors: Senior  physicians (faculty  members) | **Evaluation strategy and results**  Online questionnaire (quantitative data from mentees). High response rate; 68% (104 of 152)  Results:   - Support in career decisions by counseling - Improved networking - Increased social support - Reduced stress experience |
| **Goldstein et al., 2005. University of Washington School of Medicine, USA.** | | | |
| **Aims**   - Continuous monitoring of the student's progress in medical school | **Structure and content**  Mentoring as part of “the College system”.  Each mentor was assigned 6 medical students. | **Participants**  Mentees: A cohort of  medical students over  four years  Mentors: Senior  physicians (faculty  members) | **Evaluation strategy and results**  Evaluation by results of Mini-Clinical Evaluation Exercise  (CEX) and of Objective Structured Clinical  Examination (OSCE); students' Portfolio of  written work.  Results:   - Improved bedside skills - Improved learning skills - Evolved ability to monitor their own developmental progress |
| **Scheckler et al., 2004. University of Wisconsin Medical School, USA. Mentorship program since 1985.** | | | |
| **Aims**   - To provide role models and an opportunity for continuous professional and personal advice | **Structure and content**  Mentoring as part of “Class Mentoring Program”.  The mentors were selected by the Associate Dean for Students based on suggestions from current class mentors, chairs of departments, faculty members or volunteers. One mentor was dedicated to each class of new medical students. Each mentor had to dedicate 50% of their time to the mentorship effort. | **Participants**  Mentees: MS-1 to MS-4  Mentors: Experienced  physicians (faculty  members) | **Evaluation strategy and results**  No systematic evaluation. The authors provide a collection of qualitative statements from participants.   - Increased awareness of possibilities in professional and extraprofessional concerns - Comprehensive educational experience - Feeling of being psychologically supported |
| **Woessner et al., 1998. University of the Saarland, Germany.** | | | |
| **Aims**   - To develop personal contact between medical students and professors - To provide advice regarding study-related and private matters | **Structure and content**  One mentor was allocated up to 12 medical students.  The mentors provided their services voluntarily and received no financial compensation. They received no formal training.  The students had the opportunity to establish personal contact with the mentor, who responded to questions and problems concerning the student's study program and their private life. Joint recreational activities, like visits to restaurants, were part of the program. The mentorship program was supposed to be open to all kinds of issues or activities. | **Participants**  Mentees: Medical students of different study years  Mentors: Faculty members | **Evaluation strategy and results**  Questionnaire (quantitative and qualitative data from mentees and mentors). No data on response rate.  Results:   - High level of satisfaction; 84% of the students were very satisfied or satisfied with their mentors - All mentees wanted to extend the duration of the program - 87% of the mentors wished to continue the program - The mentors’ comments were mainly positive, emphasizing personal contact - The negative comments were mainly regarded to time pressure |
